# Molecular genetic screening of full-term small for gestational age

**DOI:** 10.1186/s12887-023-04030-0

**Published:** 2023-05-05

**Authors:** Shuman Zhang, Lingna Zhou, Lin Zhang, Yu Wang, Huaiyan Wang

**Affiliations:** 1 Department of Neonatology, Changzhou Maternity and Child Health Care Hospital, Changzhou Medical Center, Nanjing Medical University, Changzhou, Jiangsu Province China; 2 Department of Medical Genetics, Changzhou Maternity and Child Health Care Hospital, Changzhou Medical Center, Nanjing Medical University, Changzhou, Jiangsu Province China

**Keywords:** Small for gestational age, Newborn screening, Tandem mass spectrometry, Genomic sequencing, Next-generation sequencing

## Abstract

**Objective:**

To examine the clinical application of genomic screening in newborns small for gestational age (SGA), hoping to provide an efficient technique for early discovery of neonatal diseases, which is necessary to elevate survival rates and the quality of life in infants.

**Methods:**

Totally 93 full-term SGA newborns were assessed. Dried blood spot (DBS) samples were obtained at 72 h after birth, and tandem mass spectrometry (TMS) and Angel Care genomic screening (GS, using Targeted next generation sequencing) were carried out.

**Results:**

All 93 subjects were examined by Angel Care GS and TMS. No children showing inborn errors of metabolism (IEM) were detected by TMS, while 2 pediatric cases (2.15%, 2/93) were confirmed as thyroid dyshormonogenesis 6 (TDH6) by Angel Care GS. Additionally, 45 pediatric cases (48.4%) had one or more variants conferring a carrier status for recessive childhood-onset disorders, with 31 genes and 42 variants associated with 26 diseases. The top three gene-related diseases with carrier status were autosomal recessive deafness (DFNB), abnormal thyroid hormone and Krabbe disease.

**Conclusions:**

SGA is tightly associated with genetic variation. Molecular Genetic Screening allows early detection of congenital hypothyroidism and may be a potent genomic sequencing technique for screening newborns.

## Introduction

Small for gestational age (SGA) is reflected by a birthweight (BW) that is 2 standard deviations lower than the 10th percentile BW at the same gestational age. SGA is mainly due to fetal, maternal and environmental abnormalities that result in fetal growth restriction (FGR) [1, 2]. Full-term SGA (FT-SGA) infants constitute a special group of SGA infants with BW less than 2500 g at gestational age ≥ 37 weeks and < 42 weeks. FT-SGA contributes to delayed or abnormal long-term child neurodevelopment [3]. FT-SGA infants have more sophisticated appearance and less subcutaneous fat compared with normal-weight term infants. Additionally, they are prone to complications, including neonatal polycythemia, neonatal hypoglycemia, neonatal metabolic acidosis, hypomagnesemia and hypothyroidism [4–6].

In recent years, perinatal health care and the nutritional status of pregnant women in China have improved significantly, but FT-SGA infants are common in clinic. So far, FT-SGA infants are at high risk of death, non-communicable diseases and growth retardation [6]. Therefore, FT-SGA still deserves substantial attention. It is generally recognized that genetic and environmental factors contribute to the pathogenesis of FT-SGA. Recently, genomic sequencing screening of newborns has become an innovative technology and is being used in the clinical setting.

In the past, the traditional screening method was used with obvious shortcomings, including low accuracy and a high false positive rate. Then, along with the progress of detection methods, newborn screening (NBS) by tandem mass spectrometry (TMS) was developed. This method reduces the false positive rate of the traditional method, but is still not entirely accurate. Therefore, after continuous exploration, newborn genomic sequencing (NGS) is more widely used in detection assays, greatly improving data accuracy with a false positive rate close to zero.

NGS, used to detect neonatal genetic metabolic diseases, reduces the rate of misdiagnosis, and promotes the early diagnosis and treatment of diseases. So NGS is worth popularizing and applied clinically. NGS has been notably examined by the BabySeq Project [7, 8] and the North Carolina New-born Exome Sequencing for Universal Screening (NC NEXUS) study [9]. These investigators incorporated ill newborns in neonatal intensive care units (NICUs) as well as healthy newborns. They both reported newborns in the NICU are very susceptible to genetic variation, suggesting the advantage and potential of genomic screening in newborn screening. The presently applied technologies of GS mainly include whole genome sequencing (WGS), gene panel sequencing and exome sequencing (ES). Although these techniques provide accurate diagnosis, they also have limitations, including complex experiments, high cost and difficult standardization. In addition, genomic screening has not been carried out in dried blood spots (DBSs) from SGA infants.

The purpose of this study was to investigate the Angel Care genomic screening (GS, based on Targeted next generation sequencing) of DBSs from SGA infants, and to provide a basis for the clinical application of Angel Care GS.

## Materials and methods

### Study design and participants

This trial had approval from the ethics committee of Changzhou Maternal and Child Health Care Hospital. From November 2019 to December 2020, totally 93 FT-SGA newborns with NBS were recruited in this study. Exclusion criteria were: pregnant women with infections; newborns with severe malformations.

### Newborn screening by tandem mass spectrometry (TMS)

DBS specimens were obtained from the infants on 903 filter papers (Wallace Oy, Finland) 72 h after birth [10]. The DBS samples were assessed by MS/MS with NeoBase™ Non-derivatized MS/MS Kit (PerkinElmer, Finland). Cases with positive results were further assessed for clinical signs and symptoms, individualized assistant examinations and gene detection methods. Thyroid Stimulating Hormone (TSH) levels were also measured in DBSs by time-resolved fluorescence analysis (auto DELFIA 1235, Perkin Elmer). TSH ≥ 9 mIU/L was considered elevated.

### Angel care panel design

The Angel Care panel (BGI) covers the coding sequences of 159 disease-related genes, e.g., inherited metabolic diseases, endocrine diseases, hearing impairment, neuromuscular diseases, hematologic pathologies, and immune diseases [11].

### Targeted next generation sequencing

Genomic DNA was extracted with MagPure Blood & Tissue DNA KF Kit B (Magen, China), followed by DNA fragmentation with restriction enzyme (Universal Plus Fragmentation Module, VAHTS) and magnetic-bead sieving of 200-250-bp fragments, adapter ligation, PCR and library construction. A FLUOstar microplate reader (Omega) was utilized for DNA quantitation, and IDT xGen Lockdown probes were employed for capturing target regions via hybridization, prior to library pooling and quantitation. Single-strand circularization and rolling-circle replication were then performed and DNA-nanoballs were prepared for sequencing on a MGISEQ-2000 (PE100 + 10).

### Data analysis

An automated pipeline (BGI) was utilized for analyzing sequencing data. The filtered reads underwent alignment to the human reference genome (GRCh37/hg19) with Burrows-Wheeler Aligner (BWA), calling SNV and small indels below 20 bp with Genome Analysis Toolkit (GATK). The copy number variants (CNVs) of DMD exons, frequent CNVs associated with thalassemia and SMN1 exon 7 deletion were obtained as reported in a previous study [12].

The variants were: (1) 10,136 variants in a neonate-specific database (V2021.6, BGIPhoenix Database), e.g., pathogenic/likely pathogenic variants categorized based on American Society of medical genetics and genomics (ACMG) standards and guidelines; (2) low-frequency null variants not found in the database. Null variants were frameshift, stop-loss and splice donor/acceptor variants, and those losing 2 exons or more; rare variants had a frequency of ≤ 1% in both GnomAD and 1000G.

In case the variants had specific inheritance patterns of associated diseases, the newborn was considered a suspicious case: ≥1 heterozygous variants in autosomal dominant diseases; homozygous variants or 2 heterozygous variants in trans in autosomal recessive diseases; hemizygous or heterozygous variants in X-linked dominant diseases; hemizygous/homozygous variants or 2 heterozygous variants in trans in X-linked recessive diseases; variants in mitochondria-associated diseases. Variants not following a specific inheritance pattern indicated a negative specimen in genetic screening.

## Results

Totally 93 children were finally included and underwent Angel Care next generation sequencing and TMS neonatal screening 72 h following birth. As shown in Table 1, they included 45 males, and their birth weight were 2297.61 ± 64.35 g, with gestational age of 37 ± 1.08 weeks, TSH value of 3.13 ± 1.08 mIU/L. In the 93 FT-SGA subjects, NGS showed that 2 subjects [case 1 (girl) and case 2 (boy)] had suspected a genetic disease, thyroid dyshormonogenesis 6 (TDH6), as shown in Table 2. However, the TMS results were not significantly abnormal. In addition, possible pathogenic mutations in *DUOX2* were detected in both cases. The results showed a homozygous *DUOX2* variant (c.2048G > T) in case 1 and compound heterozygous mutation *DUOX2* variant (c.1588 A > T and c.2654G > T) in case 2.

**Table 1:** Baseline demographic and clinical characteristics of full-term small for gestational age (FT-SGA) infants

	n	Sex(male)	Gestational age(weeks)	Birth weight(g)	TSH (mIU/L)
All case	93	45	37.84 ± 1.08	2297.61 ± 64.35	3.13 ± 1.89

Normal distribution data are represented as mean ± SD.

**Table 2:** Genetic test results of 2 positive cases with Thyroid Dyshormonogenesis 6

Case	Sex	Mode	Gene	Exon/ Intron	cDNA change	Genotype	ACMG classificationa
Case1	Female	AR	*DUOX2* (NM_014080.4)	EX17	c.2048G > T (p.Arg683Leu)	Hom	LP
Case2	Male	AR	*DUOX2* (NM_014080.4)	EX14	c.1588 A > T (p.Lys530*)	Het	P
				EX20	c.2654G > T (p.Arg885Leu)	Het	LP

Abbreviations: AR, autosomal recessive disorder; Het, heterozygous; Hom, homozygote; P, pathogenic; LP, likely pathogenic

**Fig. 1:**
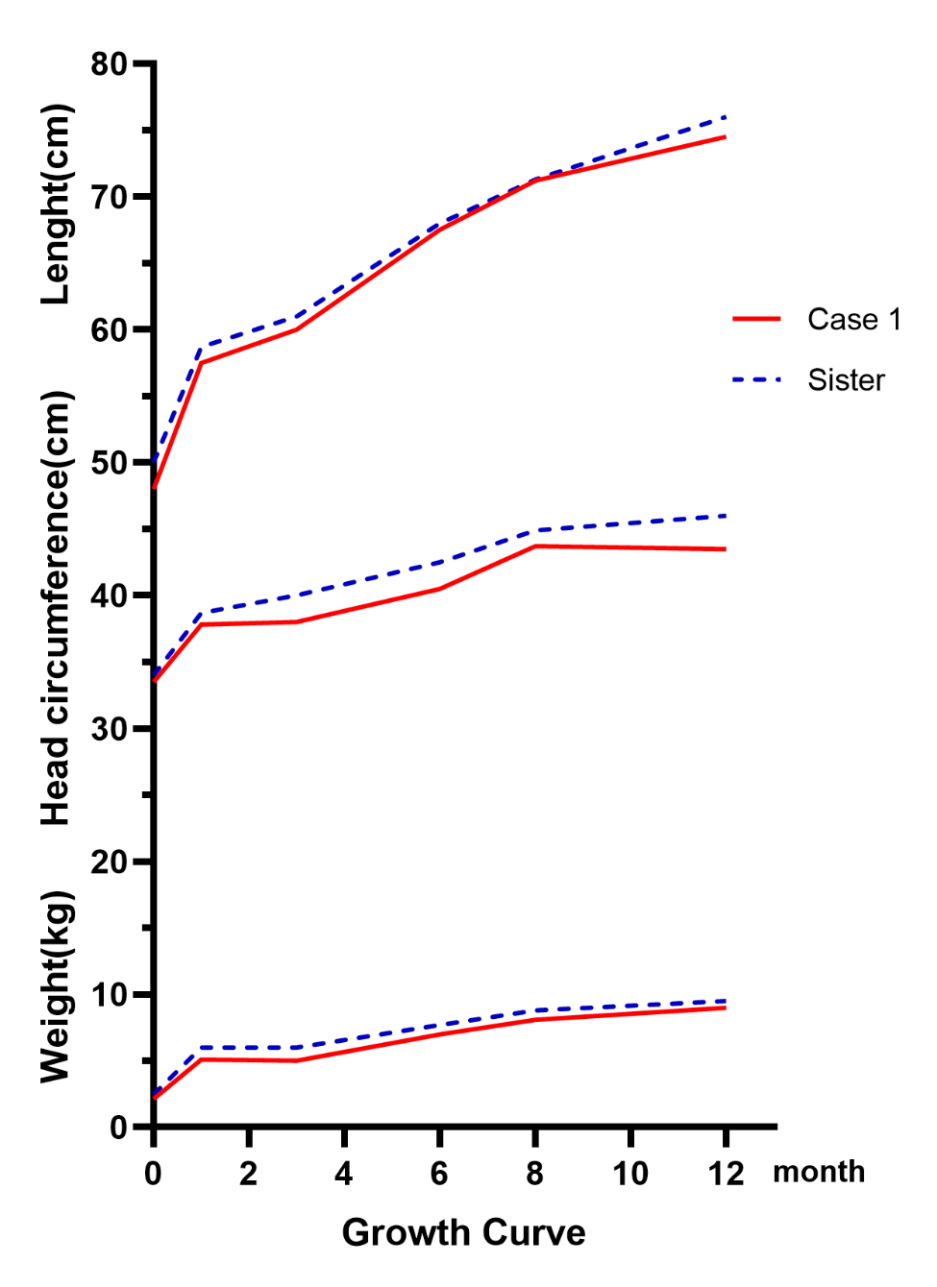
Growth curve of case 1 and her elder twin sister. The Y-axis represents weight(kg), head circumference(cm) and lenght(cm); and the X-axis represents months of age

Case 1 (girl) was born at GA 37^+ 2^ weeks and weighed 2120 g at birth, indicating a lower weight than her elder twin sister with a BW of 2480 g. She was confirmed with homozygous *DUOX2* variant in c.2048G > T (p.Arg683Leu) by Angel Care GS. Her clinical manifestations were low weight and reduced head circumference (Fig. 1), as well as less milk intake, difficulty in consuming complementary food, constipation and persistent eczema. And her TSH value by TMS was 5.25 mIU/L which was normal. Case 2 (boy) was born at 39^+ 4^ weeks of gestation with a BW of 2450 g. He was also confirmed with TDH6 through Angel Care GS with two heterozygous pathogenic mutations, including c. 1588 A > T (Lys530*) and c.2654G > T (p.Arg885Leu) in *DUOX2*. His TSH value by TMS was 10mIU/L, and the review value was was 6.12mIU/L which was also normal. Unfortunately, he was lost to follow-up because of the migrating population. In this study, another 5 infants (5/93) were had the carrier status variant in *DUOX2* as examined by GS.

The study reported 31 genes and 42 variants associated with 26 diseases in 45 SGA infants. Based on the guidelines of ACMG [13], of the 42 carrier status variants, 18 (41.9%), 19 (44.2%) and 6 (13.9%) were pathogenic (P), likely pathogenic (LP) and variants with uncertain significance (VUS), respectively. Among the 45 SGA infants, 6 had 2 variants, 3 had 3 variants, and 2 had 4 variants. Table 3 lists common genes and variants of carrier status. The top three gene-related diseases were autosomal recessive deafness (DFNB, 15/45), abnormal thyroid hormone (6/45) and Krabbe disease (KD, 4/45).

**Table 3:** Pathogenic Genes, Variants and Diseases in Present Study

Diseases	n	Genes	Exon/ Intron	cDNA Change (Amino Acids Change)	n	ACMG Classification
Autosomal recessive deafness	15	*GJB2*(NM_004004.5)	EX2E	c.109G > A(p.Val37Ile)	9	P
			EX2E	c.235delC(p.Leu79Cysfs*3)	3	P
		*TMC1*(NM_138691.2)	IVS14	c.1030-2 A > C	1	VUS
		*MYO15A*(NM_016239.3)	EX2	c.1047 C > A(p.Tyr349*)	1	LP
		*TMPRSS3*(NM_024022.2)	EX9	c.916G > A(p.Ala306Thr)	1	P
Abnormal thyroid hormone	6	*DUOX2*(NM_014080.4)	IVS28	c.3693 + 1G > T	1	P
			EX20	c.2654G > T(p.Arg885Leu)	1	LP
			EX18	c.2202G > A(p.Trp734*)	1	LP
			EX14	c.1588 A > T(p.Lys530*)	1	P
			IVS5	c.514-1G > A	1	LP
		*DUOXA2*(NM_207581.3)	EX5	c.738 C > G(p.Tyr246*)	1	VUS
Krabbe disease	4	*GALC*(NM_000153.3)	EX16	c.1901T > C(p.Leu634Ser)	2	P
			EX14	c.1630G > A(p.Asp544Asn)	1	P
			EX8	c.908 C > T(p.Ser303Phe)	1	LP
Gitelman syndrome	3	*SLC12A3*(NM_000339.2)	EX14	c.1732G > A(p.Val578Met)	3	LP
Hyperprolinemia type 1	3	*PRODH*(NM_016335.4)	EX12	c.1322T > C(p.Leu441Pro)	3	P
Tyrosine hydroxylase deficiency	3	*TH*(NM_199292.2)	IVS7	c.788 + 1G > C	2	VUS
			EX7	c.746 C > T(p.Pro249Leu)	1	LP
Cerebrotendinous xanthomatosis	3	*CYP27A1*(NM_000784.3)	IVS7	c.1263 + 1G > A	2	P
			EX9E	c.1591delT(p.Cys531Alafs*59)	1	VUS
Hepatolenticular degeneration	3	*ATP7B*(NM_000053.3)	EX16	c.3443T > C(p.Ile1148Thr)	1	LP
			EX8	c.2333G > T(p.Arg778Leu)	1	P
			EX11	c.2605G > A(p.Gly869Arg)	1	LP
Spinal muscular atrophy	2	*SMN1*(NM_000344.3)	EX7	EX7 DEL	2	P
Familial mediterranean fever	2	*MEFV*(NM_000243.2)	EX3	c.928_942delGCTGCGAGTCCCCGCinsT(p.Ala310Leufs*10)	2	LP
Familial hemophagocytic lymphohistiocytosis type 3	2	*UNC13D*(NM_199242.2)	EX32E	c.3229_3235delCGGGCCA(p.Arg1077Serfs*48)	2	VUS
Citrin deficiency	2	*SLC25A13*(NM_014251.2)	EX9	c.852_855delTATG(p.Met285Profs*2)	2	P
Methylmalonic acidemia	2	*MMACHC*(NM_015506.2)	EX4E	c.567dupT(p.Ile190Tyrfs*13)	1	P
		*MMUT*(NM_000255.3)	EX9	c.1663G > A(p.Ala555Thr)	1	LP
BH4-deficient hyperphenylalaninemia type A	1	*PTS*(NM_000317.2)	EX3	c.169_171delGTG(p.Val57del)	1	LP
COL4A4-associated Alport syndrome	1	*COL4A4*(NM_000092.4)	EX47	c.4715 C > T(p.Pro1572Leu)	1	LP
Pendred syndrome/Autosomal recessive hereditary deafness type 4 with vestibular aqueduct enlargement	1	*SLC26A4*(NM_000441.1)	EX10	c.1226G > A(p.Arg409His)	1	P
Alpha thalassemia	1	*HBA1/HBA2*(NM_000558.3/NM_000517.4)	---	-α3.7	1	P
Phenylketonuria	1	*PAH*(NM_000277.1)	EX12	c.1238G > C(p.Arg413Pro)	1	P
Hypophos Phatasia	1	*ALPL*(NM_000478.4)	EX6	c.542 C > T(p.Ser181Leu)	1	LP
Homocystinuria due to cystathionine beta synthase deficiency	1	*CBS*(NM_000071.2)	EX6	c.502G > A(p.Val168Met)	1	LP
Tyrosinemia type 2	1	*TAT*(NM_000353.2)	EX12E	c.1297 C > T(p.Arg433Trp)	1	LP
Niemann-Pick disease type A/B	1	*SMPD1*(NM_000543.4)	EX2	c.995 C > G(p.Pro332Arg)	1	LP
Glycogen storage disease type Ib/Ic	1	*SLC37A4*(NM_001164277.1)	EX9	c.1042_1043delCT(p.Leu348Valfs*53)	1	LP
Primary Coenzyme Q10 deficiency type 7	1	*COQ4*(NM_016035.3)	IVS4	c.402 + 1G > C	1	P
Primary carnitine deficiency	1	*SLC22A5*(NM_003060.3)	EX8	c.1400 C > G(p.Ser467Cys)	1	P
Medium chain acyl-CoA dehydrogenase deficiency	1	*ACADM*(NM_000016.4)	EX11	c.1085G > A(p.Gly362Glu)	1	LP

Abbreviations: P, pathogenic; LP, likely pathogenic; VUS, variant of uncertain significance

## Discussion

In 2010, about 32.4 million SGA newborns (27% of live births) were born in low- and middle-income nations. Among them, 10.6 million were FT-SGA [14]. China had an incidence of SGA of approximately 6.5% [14], ranking among the top 5 countries in the world. A recent survey showed a prevalence of SGA of about 12.28% in Guangdong Province, China [15]. Therefore, special attention should be paid to SGA because of its high incidence.

More and more studies have shown that SGA may also be caused by gene polymorphism and mutation [16–18]. However, no large sample trials with DBSs in SGA have been carried out. Therefore, we applied the novel genome sequencing project (“Angel Care”) utilizing the GS technology in FT-SGA, which detected 159 disease-associated genes.

By Angel Care GS, we detected two heterozygous and one homozygous pathogenic mutations in the *DUOX2* gene which associated with TDH6 and congenital hypothyroidism (CH). It was previously demonstrated *DUOX2* is the commonest gene mutation in Chinese pediatric CH cases [19], and a high degree of phenotypic heterogeneity was observed [20]. Recently, we hypothesized that CH may lead to abnormal fetal growth and development, which not only is manifested at birth, but also becomes more serious through epigenetic regulation, leading to permanent physiological and metabolic changes. In the long run, even in adulthood, this may cause mental retardation and underdevelopment, which may last for generations. Early detection, diagnosis and treatment is necessary to improve infants’ survival and quality of life.

Currently, diagnostic and treatment methods for CH are established, and NBS can be utilized for early CH diagnosis by detecting TSH and FT4. However, in SGA infants, TSH levels surge lightly after birth, and the initial serum total FT4 concentrations are lower compared with those of normal term infants, leading to a decrease during the first few days after birth rather than an increase as seen in normal term infants [21]. These differences may contribute to the missed diagnosis of CH by NBS. The results of this study indicated that the Angel Care GS provides a higher rate of neonatal disease screening compared with NBS.

In addition, valuable carrier information and cases of genetic disease carrier status were detected in this study. The genetic disease with the highest number of valuable carriers was DFNB (15/45, 33.3%). Detecting neonatal hearing abnormality early may reduce the negative effects on speech and language functions. Appropriate tests to screen for hearing impairment are available, including Otoacoustic Emission and Auditory Evoked Potential. However, there is a lack of uniformity in protocols adopted within newborn hearing screening [22]. On the other hand, ≥ 50% of congenital or childhood hearing loss cases involve genetic factors. In non-syndromic hearing loss, accounting 70% of all genetic hearing loss, about 80%, 15% and 1–2% of cases are autosomal recessive, autosomal dominant, and mitochondrial or X-linked, respectively. Currently, > 140 deafness-associated genes are known. Reports assessing such genes provided great molecular insights into the inner ear’s functions [23]. Mutations in the GJB2 gene drive up to 50% of pre-lingual, recessive deafness [24]. In this study, the most heterozygous pathogenic mutations in GJB2 was c.109G > A (p.Val37Ile), which has been confirmed to be pathogenic for Autosomal Recessive Deafness 1 A (DFNB1A) [25, 26]. The carriers are currently undiagnosed but may be more susceptible to hearing loss in childhood and even adulthood.

The genetic disease with the second highest number of valuable carriers was abnormal thyroid hormone (6/45, 13.3%). These variants of carrier status were located in *DUOX2* and *DUOXA2* Genetic factors, in particular mutations in *DUOX2* and *DUOXA2* is a major contributor to the overall increase in the incidence of CH and transient congenital hypothyroidism (TCH) [27, 28]. Therefore, the neonatal *DUOXA2* gene variant should also be of concern to us. Another genetic disease with valuable carriers, KD is an autosomal recessive lysosomal disease and characterized by neurodegeneration, whose severity depends on the age of onset. Consistent with the results of our study in Table 3, the genetic loss of function of the lysosomal enzyme galactocerebrosidase (GALC) leads to KD. And the incidence of KD by newborn screening in USA was 1 in 55,000 to 418,000 births [29]. Up to now, hematopoietic cell transplantation is the only currently available treatment of infants with KD with high healthcare costs [30]. Especially in infantile KD patients, earlier treatment may mean better outcome. Therefore, early screening may improve the prognosis of children with KD.

As a consequence, this study provides critical insights, which could allow personalized and correct genetic counseling. These data may help plan future treatment management, particularly in reproductive planning. In fact, whether reporting carrier data to the newborn’s parents is necessary remains controversial.

At the same time, the current research showed no correlation between genetic variation and SGA infants. This may require further clinical and genetic analyses. Traditional screening methods have low specificity and sensitivity, making it difficult to identify genetic and metabolic diseases. However, the specificity and sensitivity of gene screening have been significantly improved, which could markedly decrease false positives, fundamentally solving the limitations of traditional screening methods, and promote the healthy development of children. The current study also had the following limitation: the sample size was statistically insufficient, and in-depth research is needed.

In conclusion, positive neonatal diseases missed by NBS with TMS and disease-related mutated gene carriers were identified in this study by Angel Care GS, providing a more effective method for disease screening in SGA which need further and more studies. In addition, Angel Care GS may be beneficial for long-term health care of SGA infants.

## Data Availability

The datasets generated and analysed during the current study are not publicly available due Regulations on the management of human genetic resources in China but are available from the corresponding author on reasonable request.

## Abbreviations

SGA: Small for gestational age
DBSs: Dried blood spots
TMS: Tandem mass spectrometry
NBS: Newborn screening
TMS: Tandem mass spectrometry
GS: Genomic screening
TDH6: Thyroid dyshormonogenesis 6
DFNB: Autosomal recessive deafness
BW: Birth weight
FGR: Fetal growth restriction
FT-SGA: Full-term SGA
NICUs: Neonatal intensive care units
WGS: Whole genome sequencing
ES: Exome sequencing
TSH: Thyroid Stimulating Hormone
CH: Congenital hypothyroidism
KD: Krabbe disease

## References

[1] Campisi SC, Carbone SE, Zlotkin S (2019). Catch-Up growth in full-term small for gestational age infants: a systematic review. Advances in nutrition (Bethesda. Md).

[2] Saggese G, Fanos M, Simi F. SGA children: auxological and metabolic outcomes - the role of GH treatment. The journal of maternal-fetal & neonatal medicine: the official journal of the European Association of Perinatal Medicine, the Federation of Asia and Oceania Perinatal Societies, the International Society of Perinatal Obstet 2013, 26 Suppl 2:64–7. 10.3109/14767058.2013.832870 24059556

[3] Heinonen K, Räikkönen K, Pesonen AK, Andersson S, Kajantie E, Eriksson JG, Wolke D, Lano A (2010). Behavioural symptoms of attention deficit/hyperactivity disorder in preterm and term children born small and appropriate for gestational age: a longitudinal study. BMC Pediatr.

[4] Rovet JF. Congenital hypothyroidism: an analysis of persisting deficits and associated factors. Child neuropsychology: a journal on normal and abnormal development in childhood and adolescence 2002, 8(3):150–62. 10.1076/chin.8.3.150.13501 12759831

[5] Ewing AC, Ellington SR, Shapiro-Mendoza CK, Barfield WD, Kourtis AP (2017). Full-term small-for-gestational-age Newborns in the U.S.: characteristics, Trends, and morbidity. Matern Child Health J.

[6] Franco B, Laura F, Sara N, Salvatore G (2013). Thyroid function in small for gestational age newborns: a review. J Clin Res Pediatr Endocrinol.

[7] Holm IA, Agrawal PB, Ceyhan-Birsoy O, Christensen KD, Fayer S, Frankel LA, Genetti CA, Krier JB, LaMay RC, Levy HL (2018). The BabySeq project: implementing genomic sequencing in newborns. BMC Pediatr.

[8] Wojcik MH, Zhang T, Ceyhan-Birsoy O, Genetti CA, Lebo MS, Yu TW, Parad RB, Holm IA, Rehm HL, Beggs AH (2021). Discordant results between conventional newborn screening and genomic sequencing in the BabySeq Project. Genet medicine: official J Am Coll Med Genet.

[9] Milko LV, Rini C, Lewis MA, Butterfield RM, Lin FC, Paquin RS, Powell BC, Roche MI, Souris KJ, Bailey DB (2018). Evaluating parents’ decisions about next-generation sequencing for their child in the NC NEXUS (North Carolina Newborn Exome sequencing for Universal Screening) study: a randomized controlled trial protocol. Trials.

[10] Yang Y, Wang L, Wang B, Liu S, Yu B, Wang T (2019). Application of next-generation sequencing following Tandem Mass Spectrometry to Expand Newborn Screening for Inborn errors of metabolism: a Multicenter Study. Front Genet.

[11] Yang Y, Wang Y, Zhou L, Long W, Yu B, Wang H. Molecular Genetic Screening of Neonatal Intensive Care Units: Hyperbilirubinemia as an Example. The application of clinical genetics 2022, 15:39–48. 10.2147/TACG.S362148 PMC9124469 35611242

[12] Shang X, Peng Z, Ye Y, Asan, Zhang X, Chen Y, Zhu B, Cai W, Chen S, Cai R (2017). Rapid targeted Next-Generation sequencing platform for Molecular Screening and clinical genotyping in subjects with hemoglobinopathies. EBioMedicine.

[13] Riggs ER, Andersen EF, Cherry AM, Kantarci S, Kearney H, Patel A, Raca G, Ritter DI, South ST, Thorland EC (2020). Technical standards for the interpretation and reporting of constitutional copy-number variants: a joint consensus recommendation of the American College of Medical Genetics and Genomics (ACMG) and the Clinical Genome Resource (ClinGen). Genetics in medicine: official. J Am Coll Med Genet.

[14] Lee AC, Katz J, Blencowe H, Cousens S, Kozuki N, Vogel JP, Adair L, Baqui AH, Bhutta ZA, Caulfield LE (2013). National and regional estimates of term and preterm babies born small for gestational age in 138 low-income and middle-income countries in 2010. The Lancet Global health.

[15] He H, Miao H, Liang Z, Zhang Y, Jiang W, Deng Z, Tang J, Liu G, Luo X. Prevalence of small for gestational age infants in 21 cities in China, 2014–2019. 2014-03-27 2021, 11(1):7500. 10.1038/s41598-021-87127-9 PMC8021546 33820960

[16] Stalman SE, Solanky N, Ishida M, Alemán-Charlet C, Abu-Amero S, Alders M, Alvizi L, Baird W, Demetriou C, Henneman P (2018). Genetic analyses in small-for-gestational-age newborns. J Clin Endocrinol Metab.

[17] Freire BL, Homma TK, Funari MFA, Lerario AM, Vasques GA, Malaquias AC, Arnhold IJP, Jorge AAL (2019). Multigene sequencing analysis of children born small for gestational age with isolated short stature. J Clin Endocrinol Metab.

[18] Peeters S, Declerck K, Thomas M, Boudin E, Beckers D, Chivu O, Heinrichs C, Devriendt K, de Zegher F, Van Hul W et al. DNA Methylation Profiling and Genomic Analysis in 20 Children with Short Stature Who Were Born Small for Gestational Age. J Clin Endocrinol Metab 2020, 105(12). 10.1210/clinem/dgaa465 32685970

[19] Yu B, Long W, Yang Y, Wang Y, Jiang L, Cai Z, Wang H (2018). Newborn screening and Molecular Profile of congenital hypothyroidism in a Chinese Population. Front Genet.

[20] Long W, Zhou L, Wang Y, Liu J, Wang H, Yu B. Complicated Relationship between Genetic Mutations and Phenotypic Characteristics in Transient and Permanent Congenital Hypothyroidism: Analysis of Pooled Literature Data. International journal of endocrinology 2020, 2020:6808517. 10.1155/2020/6808517 PMC7275948 32565793

[21] Wassner AJ (2017). Pediatric Hypothyroidism: diagnosis and treatment. Paediatr Drugs.

[22] Kanji A, Khoza-Shangase K, Moroe N (2018). Newborn hearing screening protocols and their outcomes: a systematic review. Int J Pediatr Otorhinolaryngol.

[23] Yang T, Guo L, Wang L, Yu X (2019). Diagnosis, intervention, and Prevention of genetic hearing loss. Adv Exp Med Biol.

[24] Petersen MB, Willems PJ (2006). Non-syndromic, autosomal-recessive deafness. Clin Genet.

[25] Pshennikova VG, Barashkov NA, Romanov GP, Teryutin FM, Solov’ev AV, Gotovtsev NN, Nikanorova AA, Nakhodkin SS, Sazonov NN, Morozov IV (2019). Comparison of predictive in Silico tools on missense variants in GJB2, GJB6, and GJB3 genes Associated with autosomal recessive deafness 1A (DFNB1A). Sci World J.

[26] Shen J, Oza AM, Del Castillo I, Duzkale H, Matsunaga T, Pandya A, Kang HP, Mar-Heyming R, Guha S, Moyer K (2019). Consensus interpretation of the p.Met34Thr and p.Val37Ile variants in GJB2 by the ClinGen hearing loss Expert Panel. Genet medicine: official J Am Coll Med Genet.

[27] Liu R, Tian JL, Huang XL, Song YZ. Genetic Factors Causing Thyroid Dyshormonogenesis as the Major Etiologies for Primary Congenital Hypothyroidism: Clinical and Genetic Characterization of 33 Patients. J Clin Med 2022, 11(24). 10.3390/jcm11247313 PMC9786654 36555929

[28] Peters C, Schoenmakers N (2022). MECHANISMS IN ENDOCRINOLOGY: the pathophysiology of transient congenital hypothyroidism. Eur J Endocrinol.

[29] Dangouloff T, Boemer F, Servais L (2021). Newborn screening of neuromuscular diseases. Neuromuscul Disord.

[30] Ghabash G, Wilkes J, Barney BJ, Bonkowsky JL (2022). Hospitalization burden and incidence of Krabbe Disease. J Child Neurol.

